# 1-Methylxanthine enhances memory and neurotransmitter levels

**DOI:** 10.1371/journal.pone.0313486

**Published:** 2025-01-16

**Authors:** Ralf Jäger, Sidney Abou Sawan, Marco Orrú, Grant M. Tinlsey, Martin Purpura, Shawn D. Wells, Kylin Liao, Ashok Godavarthi

**Affiliations:** 1 Ingenious Ingredients L.P., Lewisville, TX, United States of America; 2 Increnovo LLC, Whitefish Bay, WI, United States of America; 3 Iovate Health Sciences International, Oakville, ON, Canada; 4 Department of Pharmacology and Toxicology, University of Utah, Salt Lake City, UT, United States of America; 5 Department of Kinesiology and Sport Management, Texas Tech University, Lubbock, TX, United States of America; 6 Radiant Research Services Pvt. Ltd., Bangalore, India; Universidade do Estado do Rio de Janeiro, BRAZIL

## Abstract

1-Methylxanthine (1-MX) is the major metabolite of caffeine and paraxanthine and might contribute to their activity. 1-MX is an adenosine receptor antagonist and increases the release and survivability of neurotransmitters; however, no study has addressed the potential physiological effects of 1-MX ingestion. The aim of this study was to compare the effect of 1-MX on memory and related biomarkers in rats compared to control. Memory (escape latency in the Morris water maze test), neurotransmitters (acetylcholine, dopamine, gamma-amino butyric acid (GABA)), and neurochemicals (BDNF, catalase, glutathione, Amyloid Beta and cyclic GMP) were analyzed from whole brain samples in young (8-weeks-old) and aged (16-months-old) rats following 12 days of supplementation (100 mg/d HED of 1-MX [UPLEVEL®, Ingenious Ingredients L.P., Lewisville, TX, USA]) via oral gavage. 1-MX supplementation reduced escape latency by 39% in young animals and 27% in aged animals compared to controls (both *p*<0.001). Additionally, 1-MX increased the levels of acetylcholine, dopamine, GABA, and cyclic GMP (all *p*<0.001). Furthermore, 1-MX supplementation led to reduced amyloid beta and higher catalase, BDNF and glutathione concentrations (*p*<0.001). Collectively, our findings suggest that 1-MX may have cognitive-enhancing and neuroprotective properties.

## Introduction

Caffeine is a stimulant when consumed acutely in high doses and re-optimizes brain functioning when consumed chronically in moderate doses, as shown in multi-omic measurements in rodents [1] and in humans [2]. Caffeine is rapidly and almost completely absorbed in the stomach and small intestine and metabolized in the liver. Caffeine undergoes demethylation catalyzed by cytochrome P450; an enzyme responsible for about 95% of caffeine metabolism. Metabolism of caffeine is influenced by numerous different factors, including age, diet, smoking, environmental factors, medications, including contraceptives, and genetics [3]. Individuals with a homogenous A allele of the CYP1A2 gene tend to produce more cytochrome P450 and consequently metabolize caffeine faster [4]. Caffeine’s main metabolite, paraxanthine (1,7-dimethylxanthine), accounts for 70–72% of caffeine ingested, and 84% of the methylxanthine metabolic by-products [5]. A minor part of caffeine is transformed to theobromine(12%, 3,7-dimethylxanthine) and theophylline (4%, 1,3-dimethylxanthine).

Paraxanthine, the primary caffeine metabolite, has been shown to possess similar [6] and/or superior [7] psychoactive properties than caffeine. We recently demonstrated that in young adults, 200 mg of paraxanthine, compared to placebo, may acutely affect short-term memory, reasoning, and response time to cognitive challenges [8], and may serve as an effective nootropic nutrient at an acute dose as low as 50 mg [9]. In a separate animal study, we demonstrated that paraxanthine enhances learning and memory to a greater extent than caffeine in young and old animals which are associated with enhanced brain-derived neurotropic factor (BDNF) in brain homogenate [10].

**Fig 1:**
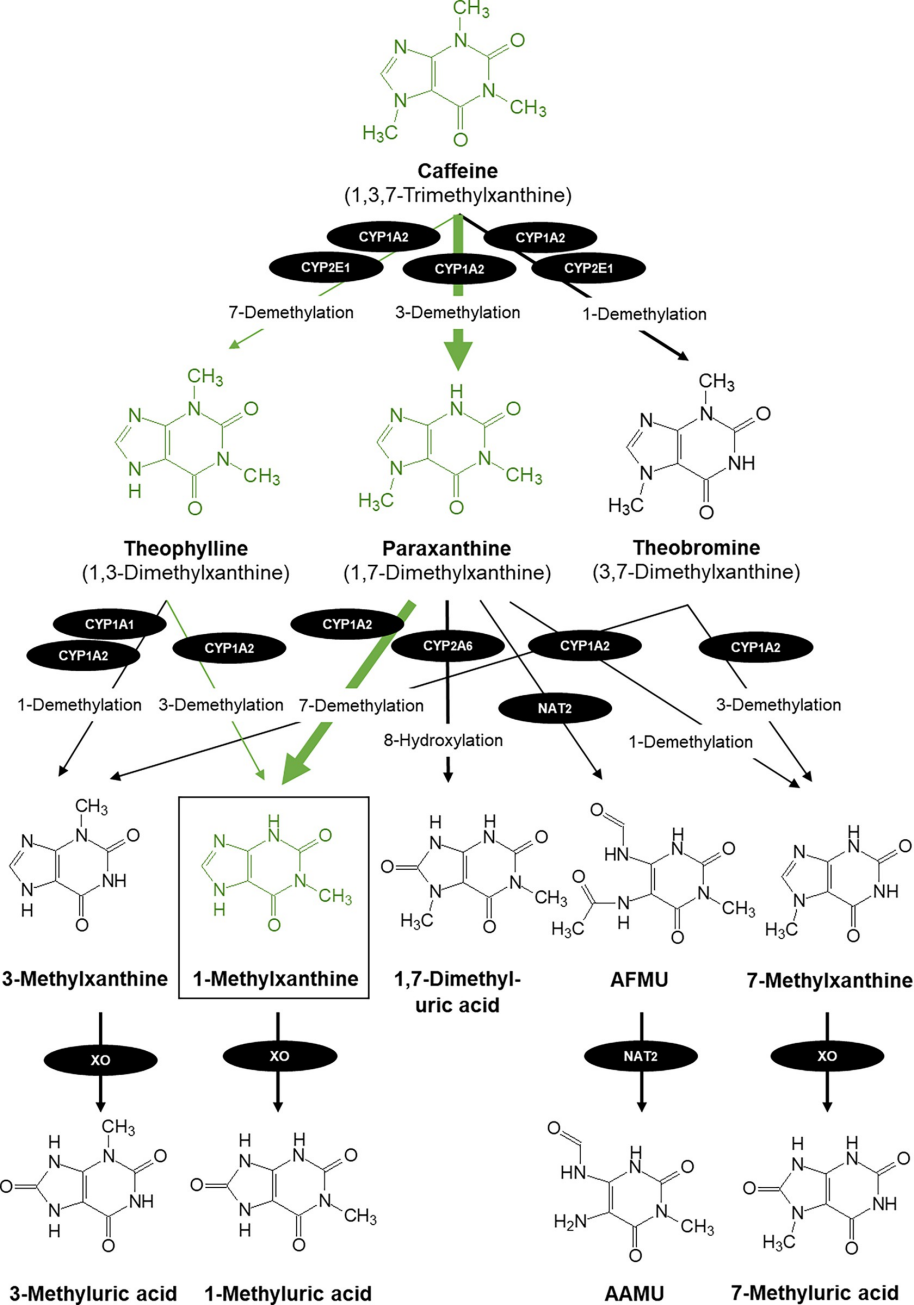
1-MX is produced through 3-demethylation of theophylline and 7-demethylation of paraxanthine. XO = xanthine oxidase; AFMU = 5-acetyl-amino-6-formylamino-3-methyluracil; AAMU = 5-acetylamino-6-amino-3-methyluracil; NAT2 = N-acetyltransferase 2; CYP1A2 = cytochrome P450 family 1 subfamily A member 2.

1-Methylxanthine (1-MX) is a metabolite of caffeine (1,3,7-trimethylxanthine) (Fig 1). 1-MX is produced through 3-demethylation of theophylline and 7-demethylation of paraxanthine. 1-MX accounts for approximately 25% of theophylline’s metabolites, while most theophylline is being transformed to 1,3-dimethyluric acid [11]. In contrast, the major metabolite of paraxanthine is 1-MX. The combined formation of paraxanthine’s 7-demethylated products and 5-acetyl-amino-6-formylamino-3-methyluracil (AFMU) was found to account for 67% of paraxanthine clearance [9]. It is estimated that a total of 40–48% of caffeine will metabolize to 1-MX with >95% of that amount via caffeine’s initial metabolism to paraxanthine [11, 12] (**Fig 1**). The ability to metabolize caffeine decreases with age. The expression of cytochrome P450 initially increase with age from its low levels in neonatal stages, however, decrease in aged animals [13, 14].

1-MX is a natural dietary ingredient that can be found in several different foods, such as garden tomatoes (*Solanum lycopersicum*), lotus (*Nelumbo*), okras (*Abelmoschus esculentus*), tamarinds (*Tamarindus indica*), chestnuts (*Castanea*), white tea (*Camellia sinensis* L.) [15, 16], cacao (*Theobroma cacao*) [17] and coffee (*Coffea eugenioides*, *Coffea bengalensis*) [18]. 1-MX is an adenosine receptor antagonist and as a major metabolite of caffeine and paraxanthine, 1-MX might contribute to their activity [19, 20]. 1-MX has also been shown to activate reticulum ryanodine receptor (RyR) channels resulting in augmentation of the excitability of neurons and thus their capacity to release neurotransmitters [21], and to improve their survival [22]. In contrast, 3- and 7-methylxanthine do not activate RyR channels [21].

Aging is accompanied by a range of neurological changes that can impact cognitive function, including declines in synaptic plasticity [23], neurogenesis [24], and alterations in neurotransmitter systems [25]–all of which may impair learning and memory. Clinical studies in young [26–28] and old [29, 30] adults, generally demonstrate that CAF enhances attention, alertness, and short-term memory in dosages ranging from ~32-500mg. Paraxanthine, a less-studied CAF metabolite, has distinct pharmacokinetic and pharmacodynamic properties [6, 12] increasing dopaminergic activity [22] and potentially improving memory consolidation more effectively than caffeine. Clinical studies demonstrate that 200 mg of paraxanthine enhances memory, reaction time, and attention for up to 6-hours in young healthy adults [8], and that acute ingestion as low as 50 mg of PXN for 7-days enhances measures of cognition, memory, reasoning, response time, and helped sustain attention [9]. However, no study has addressed the potential physiological effects of 1-MX ingestion and the impact of aging. The primary aim of this study was to determine if 1-MX ingestion affects memory in young and old rats and whether 1-MX can influence neurotransmitter levels, β-amyloid production, BDNF, and antioxidant capacity.

## Materials and methods

### Animals and study design

Sixteen male Swiss Albino rats aged 8-weeks (YOUNG) and 16-months-old (OLD) were housed in an animal room at a constant temperature (22 ± 3°C) and humidity (30–70%) under a 12:12 h light-dark cycle with standard laboratory diet (Purina 5L79, Rat and Mouse 18% protein; PMI Nutrition International, Brentwood, MO, USA). Animals were housed in a standard polypropylene cage (4 animals/cage) with stainless steel top grill having facilities for pelleted food and drinking water. Sterile corncob was used as bedding material and changed every day. Reverse osmosis (RO) purified water was provided ad libitum. All procedures involving animals were conducted humanely and were performed by or under the direction of trained personnel. The study protocol was reviewed and approved by Institutional Animal Ethical Committee (IAEC) of Radiant Research Services Pvt. Ltd (Bangalore, India, approval number RR/IAEC/104-2022, 29- Jan-2022).

**Fig 2:**
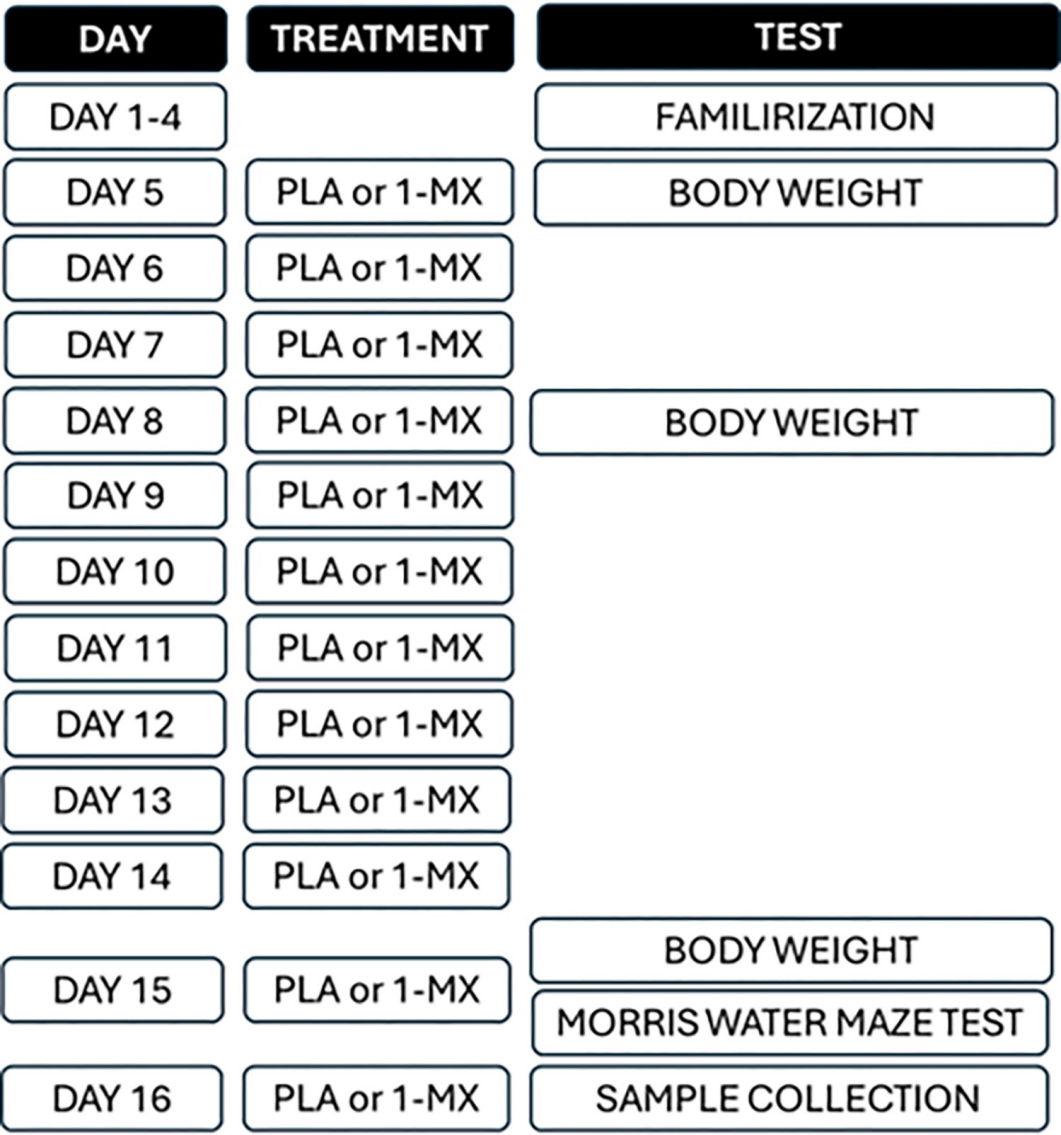
Timeline of treatments and tests.

To compare the effect of 1-MX against control (CON) and the impact of age, young and old rats were evenly divided into 2 groups per age group (n = 8 per group). The 1-MX dose administered to the rats was calculated using US Food and Drug Administration for human equivalence doses (HED), assuming a human weight of 60 kg [31]. 100 mg/d HED of 1-MX (UPLEVEL®, Ingenious Ingredients L.P., Lewisville, TX, USA), rat dose: 10.28 mg/kg/d) and 0.5% Carboxy Methyl Cellulose sodium were administered via oral gavage using disposable polypropylene syringes with sterilized stainless steel gavage tubes. The test items were administered once each day by oral route for 12 consecutive days at 9:00–11:00 hours (days 5–16). The volume administered to each rat was 10 mL/kg/day. Body weight was recorded at baseline and on days 5, 8 and 15 and the treatment dose was adjusted based on recent body weights recorded during the treatment period (Fig 2) Using the same study protocol, data involving the CON group has been previously published [10].

### The Morris water maze test

Memory was assessed using an open circular pool (Orchid Scientific & Innovative India Pvt Ltd, 183 cm inner diameter and 76 cm in height) that was filled approximately half-way with water (25 ± 1°C) [32, 33]. The interior of the pool was as close to being featureless as possible and placed in a room with geometric shapes on the wall serving as spatial cues. A platform was placed into one quadrant of the pool and submerged 1 cm below the water surface. The water was colored with non-toxic black colorant preventing the animals from seeing the platform which redirects the animals to use external maze cues. Days 1–4 served as familiarization period in which animals were trained to locate the submerged platform in a constant location. Thus, as the animals become more familiar with the task, they can find the platform more quickly highlighting spatial memory and learning. During training, a rat started at one of four starting points (North, South, East, West) and was allowed to swim until it located the platform or until 60 seconds had elapsed. If the rat left the platform within 15 seconds after reaching it, the measurement continued. After initial training the probe test was conducted by hiding the platform to ensure that the performance of animals was truly dependent on the spatial memory. After the completion of the familiarization the animals were supplemented for 10 consecutive days (Days 5–14) in a randomized, placebo-controlled fashion. On day 15, 30 minutes after administration of the treatments, animals performed the Morris water maze test. The escape latency was recorded and the mean of the four starting points were used for data analysis.

### Sample collection and neurochemical analysis

On day 16, after administration of the final treatment, all animals were euthanized by 95% CO_2_ and the brain was excised and weighed. Brain tissues were rinsed with ice cold PBS (pH 7.4) to remove the excess blood thoroughly and weighed before homogenization. Brain tissue (100 mg) was taken in 900 microliters of PBS (1:9) ratio in a glass homogenizer on ice and centrifuged at 2,000 rpm for 5 minutes. The resulting supernatant was analyzed using standard ELISA assay kits: acetylcholine (BT LAB, China; Lot No.: E0698Mo), dopamine (BT LAB, China; Lot No.: E0667Mo), brain derived neurotrophic factor (BDNF; Elabscience, USA; Lot No.: E-EL-R1235), β-amyloid (1–40) (Elabscience, USA; Lot No.: E-EL-R3030), catalase (Elabscience, USA; Lot No: E-BC-K031), glutathione (Elabscience, USA; Lot No.: KTE101106), gamma-aminobutyric acid (Fine Test, China; GABA; Lot No.: ER1707) and cyclic GMP (Fine Test, China; Lot No.: ER0831).

### Statistical analysis

All outcomes except body mass were analyzed using two-way analysis of variance (ANOVA) with group and age specified as between-subjects factors. Body mass data were analyzed separately in young and aged individuals, due to the known differences in body mass between young and aged animals, using two-way ANOVA with group as a between-subjects factor and time (day) as a within-subjects factor. When needed, statistically significant effects were followed up using pairwise comparisons with the Tukey adjustment to account for multiple comparisons. Due to the multiple ANOVA tests, *p*-values for main effects and interactions were corrected using the false discovery rate method. Relevant assumptions of ANOVA tests were examined. Homogeneity of variances was tested using Levene’s test, and normality of residuals was examined through visual examination of quantile-quantile plots. For models with within-subjects’ factors (i.e., the body mass models), the Greenhouse-Geisser corrected was used if sphericity violations occurred. After *p*-value adjustments as described, statistical significance was accepted at p<0.05. Data analysis was performed using R (v. 4.2.1) and the *afex* (v. 1.1–1) and *emmeans* (v. 1.8.1–1) packages.

## Results and discussion

### Effect of 1-MX and age on body and brain weight

The data for the control group for all measures has been presented previously [10]. A main effect of day, irrespective of group, was observed for body weight in both young and aged animals, with increasing values over time (Table 1). A main effect of age, irrespective of group, was observed for brain weight, with higher values in aged animals (Table 2).

**Table 1:** Changes in body weight.

							P-values		
Age	Group	Day	Mean	SD	Median	IQR	Group	Day	Group × Day
Young	Control	0	162.91	1.71	163.30	2.58	0.51	<0.001*	0.82
		5	168.04	1.63	168.45	2.58			
		8	171.03	1.69	171.25	2.15			
		15	182.36	1.44	182.60	2.35			
	1-MX	0	163.68	1.98	163.80	2.95			
		5	168.98	1.97	168.85	2.68			
		8	172.35	1.96	171.95	1.93			
		15	183.64	1.93	183.15	2.23			
Aged	Control	0	490.60	2.00	490.20	1.35	0.52	<0.001*	0.77
		5	493.64	2.15	493.60	1.60			
		8	495.09	2.12	495.20	1.73			
		15	500.01	2.17	499.70	1.23			
	1-MX	0	490.05	2.11	490.85	2.98			
		5	492.99	2.32	493.65	3.30			
		8	494.28	2.28	495.10	3.48			
		15	498.98	2.12	498.90	2.43			

**Table 2:** 1-MX significantly changed various markers of health.

							P-values		
Variable	Group	Age	Mean	SD	Median	IQR	Group	Age	Group × Age
Brain Weight (g)	Control	Aged	1.97	0.05	1.96	0.09	0.82	<0.001*	0.92
	Control	Young	1.72	0.03	1.73	0.06			
	1-MX	Aged	2.00	0.07	1.99	0.05			
	1-MX	Young	1.73	0.03	1.74	0.04			
BDNF (pg/mL)	Control	Aged	732.16	16.51	725.63	21.32	<0.001*	<0.001*	0.18
	Control	Young	775.04	29.60	769.85	48.40			
	1-MX	Aged	793.81	33.85	783.35	41.42			
	1-MX	Young	869.04	32.79	869.74	33.99			
Amyloid Beta 1–40 (g/mL)	Control	Aged	410.68	14.04	411.38	20.88	<0.001*	<0.001*	0.17
	Control	Young	295.09	12.39	296.28	17.62			
	1-MX	Aged	357.13	10.3	355.34	15.14			
	1-MX	Young	254.6	7.43	254.3	8.88			
Catalase (U/mL)	Control	Aged	26.16	1.03	26.04	1.78	<0.001*	0.045*	0.35
	Control	Young	27.76	1.21	27.94	1.26			
	1-MX	Aged	32.78	1.26	32.67	1.69			
	1-MX	Young	33.32	1.68	33.51	1.95			
Glutathione (ug/mL)	Control	Aged	19.74	1.14	20.02	1.97	<0.001*	<0.001*	0.029*
	Control	Young	21.85	1.36	21.60	2.08			
	1-MX	Aged	25.29	0.93	25.52	1.22			
	1-MX	Young	28.57	1.65	28.81	1.95			

### Effect of 1-MX and age on escape latency

**Fig 3:**
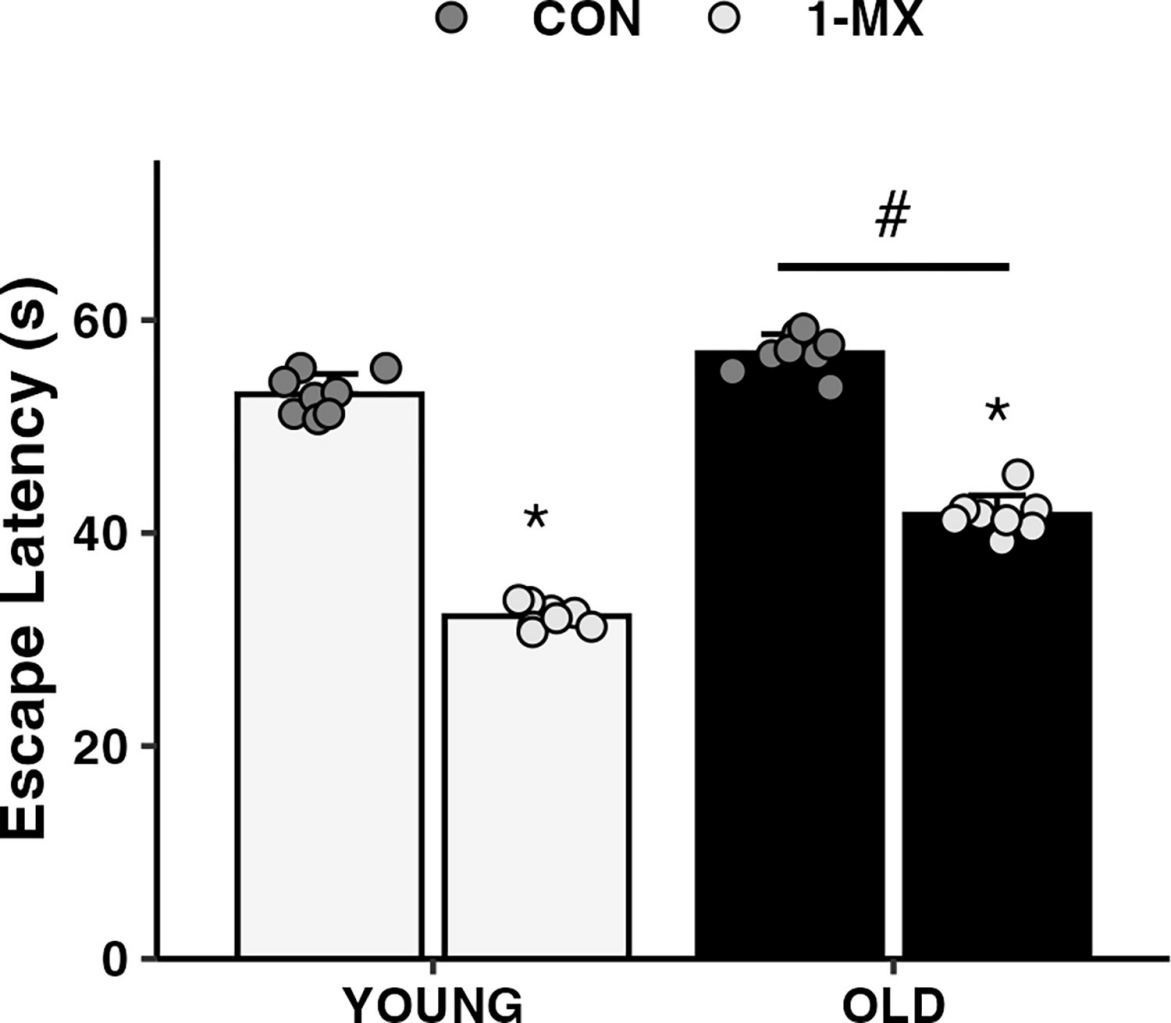
1-MX significantly improved escape latency independent of age. Separate bars are displayed for young and old rats in the 1-MX and CON conditions. Bars indicate mean responses in each group, with error bars indicating standard deviations. Points on the graph indicate individual responses. * indicates significant difference between groups within the specified age group (*p*<0.001). # indicates significant difference between ages (*p*<0.001).

A statistically significant group x age interaction was observed for escape latency (p<0.001; **Fig 3)**. Post hoc analysis revealed that 1-MX reduced escape latency in both young (by 39%) and aged (by 27%) animals, as compared to control (*p*<0.001 for both). Additionally, differences in escape latency based on age was observed for both control and 1-MX (*p*<0.001).

### Effect of 1-MX and age on neurotransmitter levels

**Fig 4:**
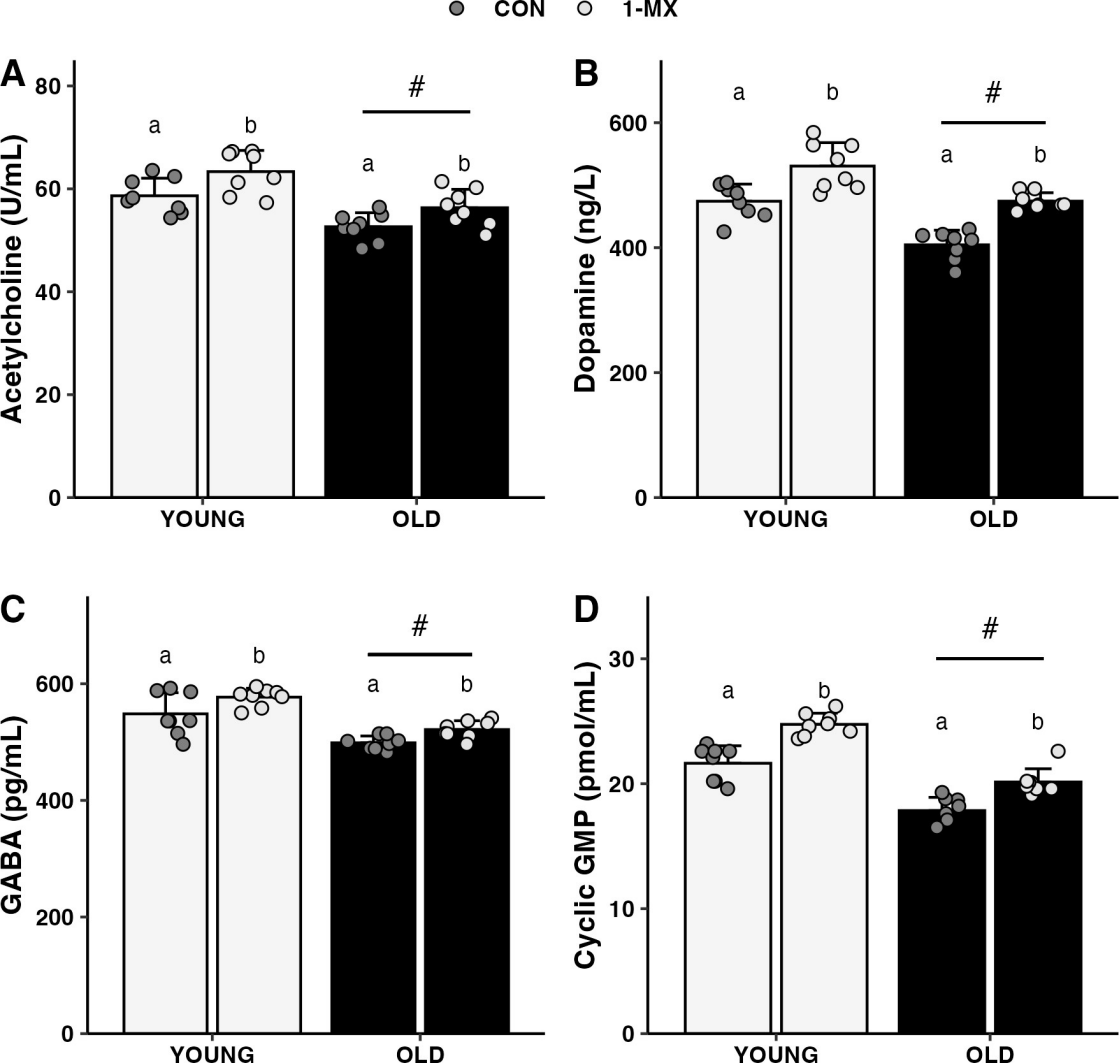
1-MX significantly increases levels of neurotransmitters. For all neurotransmitters, statistically significant effects of group and age were observed (*p*<0.01 for each), without statistically significant group × age interactions. Different letters within an age group indicate statistically different values between groups. # indicates a significant difference based on age (i.e., the age main effect).

Post-mortem neurochemical analysis showed that 1-MX increased levels of neurotransmitters after the Morris water maze test. Statistical analysis reveals that acetylcholine (OLD +7%, YOUNG +8%), dopamine (OLD +17%, YOUNG +12%), GABA (OLD +5%, YOUNG +5%), and secondary messenger cyclic GMP (OLD +13%, YOUNG +14%) levels were higher in animals supplemented with 1-MX when compared to control, *p*<0.01 for each neurotransmitter, **Fig 4**.

### Effect of 1-MX and age on markers of health

Statistically significant effects of group were observed for all physiological variables (*p* = 0.004 to *p*<0.001 for all; **Table 2**). Follow-up pairwise comparisons revealed that 1-MX differed from the control group for all outcomes. A group x age interaction was observed only for glutathione. Follow-up pairwise comparisons indicated that 1-MX had higher glutathione concentrations than control in both young and aged animals (*p*<0.001 for both). Pairwise comparisons also indicated age differences in both groups (control: *p*<0.001; 1-MX: *p* = 0.001). For all variables, statistically significant main effects of age were observed (*p* = 0.045 to *p*<0.001 for all; **Table 2**).

In this study, we explored the effects of the caffeine metabolite 1-MX on cognitive performance and neurotransmitter modulation in both young and aged animals. We demonstrate for the first that time 1-MX supplementation enhances learning and cognitive function which may be underpinned by neurotransmitter levels and antioxidative mechanisms. These data suggest that 1-MX may serve as a potential cognitive enhancer and neuroprotective properties, offering benefits across different age groups.

Our findings are in line with previous studies that show caffeine when consumed acutely in high doses improves brain function in rodents [1] and humans [2]. However, caffeine’s effectiveness can be influenced by multiple factors and is often showing diminished effects in habitual users [3]. Caffeine shows significant chronic psychoactive metabolite accumulation with daily use [34], while paraxanthine and 1-MX have mostly-inactive metabolites that are rapidly excreted [35]. Due to its hypothetical lack of chronic metabolite accumulation, paraxanthine and 1-MX, might show no diminished effects with repeated intake. Paraxanthine, the primary metabolite of caffeine, has also been shown to improve cognitive function to a greater extent than caffeine in animal models [10] and humans [36]. Younger animals typically exhibit higher levels of synaptic plasticity [21], more efficient neuronal signaling pathways [37], while aging, which is associated with declines in neurogenesis and reduced neural adaptive capacity, [22] which are related to impairments in cognitive function. Therefore, the greater reduction in escape latency among younger animals could be due to their inherently higher baseline synaptic plasticity and neuronal signaling pathways, which are more amenable to enhancement by 1-MX. In contrast, aged animals, while still benefiting significantly from 1-MX, may experience a relatively smaller improvement due to the pre-existing age-related decline in these neural processes.

1-MX has previously been shown to activate RyR channels resulting in an increased capacity to release neurotransmitters [19]. Our study confirmed the previous mechanistic findings by showing a significant increase in neurotransmitter levels in both young and aged animals following 1-MX supplementation. Acetylcholine is involved in memory and learning processes [38], and its increased availability could facilitate better synaptic transmission and cognitive function. Similarly, dopamine plays a key role in motivation and reward pathways [39], and may enhance cognitive performance by improving motivation and attentional processes. The increase in GABA, an inhibitory neurotransmitter, may help modulate neuronal excitability [40], thereby supporting cognitive function. Elevated cyclic GMP levels suggest enhanced signaling pathways, potentially leading to improved synaptic plasticity and memory formation [41]. The increases in neurotransmitter levels might be the underlying mechanism of how 1-MX increases memory. Comparatively, caffeine and paraxanthine have been shown to influence similar neurotransmitter systems, increasing dopamine and acetylcholine levels, supporting cognition. The observed increases in neurotransmitter levels with 1-MX supplementation are consistent with the mechanisms of caffeine and paraxanthine, suggesting that 1-MX may similarly enhance cognitive function by modulating key neurotransmitter systems. The discussion about neurotransmitters is at this point still speculative as the evaluation was conducted using the whole brain, and the dynamics of neurotransmitters, such as storage or turnover, were not assessed in the study.

Glutathione is an antioxidant that protects cells from oxidative stress and maintains redox homeostasis [42]. The increase in glutathione levels with 1-MX supplementation suggests that 1-MX may enhance the animals’ antioxidative defense mechanisms. This is particularly important in aged animals, where oxidative stress is typically higher due to accumulated cellular damage over time. 1-MX supplementation increased glutathione levels, which may help mitigate age-related oxidative damage, thereby preserving cognitive function and overall neuronal health. The observed increase in catalase and glutathione levels suggests that 1-MX enhances the brain’s antioxidative defenses, which is particularly important in mitigating oxidative stress associated with aging. This antioxidative effect is also observed with caffeine and paraxanthine [10], which are known to upregulate endogenous antioxidant systems [43], thereby protecting neurons from oxidative damage [44]. 1-MX-induced increases in glutathione and catalase suggest 1-MX may share similar antioxidative properties, with caffeine and paraxanthine.

Our study also found that 1-MX significantly increases other markers of brain health. Brain-derived neurotrophic factor (BDNF) levels were higher in animals supplemented with 1-MX, indicating enhanced neuronal plasticity [45] which provides a mechanism-of-action for the observed increase in memory [46]. Similar to caffeine and its primary metabolite paraxanthine, which have been shown to elevate BDNF levels [10], 1-MX appears to facilitate these neurotrophic processes. Paraxanthine has been shown to increase BDNF levels to a greater extend compared to caffeine in a preclinical study [10], which translated in increased cognitive performance in a clinical study investigating the ability to shift between tasks [34]. BDNF and GABAergic systems that are involved in critical CNS functions, ranging from neuronal development and neuronal survival to learning and memory [47, 48]. In addition, they have significant roles in neurotoxicity and neurodegenerative disease [47]. The observed increased levels in young and aged animal support improved brain health and cognitive function, and the potential long-term protective effects of optimized levels in young animals should be investigated in future studies. The reduction in amyloid beta 1–40 levels indicates a potential role for 1-MX in reducing amyloid plaque formation, which linked to the development of cognitive impairment and dementia [49, 50]. This finding is noteworthy as it parallels the protective effects of caffeine and paraxanthine, which has been shown to lower amyloid beta levels [10]. The parallels with caffeine and paraxanthine highlight the potential of 1-MX to confer similar neuroprotective and cognitive benefits, supporting its novelty to enhance cognition.

While our study provides novel insights into the cognitive-enhancing effects of 1-MX, several limitations are acknowledged. First, the study was conducted on animal models, which may not fully replicate the complexity of human cognitive processes and aging. Second, the duration of 1-MX supplementation was relatively short, and long-term effects, including dose-response relationship of 1-MX, remain unknown. Another limitation is the lack of behavioral assessments beyond escape latency, which could provide a more comprehensive evaluation of cognitive function. For example, we did not measure swim speed and distance measurements, as alterations of locomotion could be responsible for differences in escape latency. A direct comparison would provide a clearer understanding of the relative efficacy and potential advantages of 1-MX over these methylxanthines. Future research should directly compare the effects of 1-MX with tri-(caffeine), di-(paraxanthine, theophylline, and theobromine) and methylxanthines (3-MX, 7-MX) as well as their combination, as these compounds have different pharmacokinetic, safety and efficacy profiles. Finally, while our approach of male rats allowed for controlled investigation of 1-MX effects within a single sex, it limits the generalizability of our findings across both sexes. Future research should include female rats to examine potential sex differences in response to 1-MX. Future studies should expand on the potential effects of 1-MX on biomarkers like glutamate, serotonin, adenosine and cyclic AMP as well as investigate differences in neurotransmitter in specific areas of the brain, especially those involved in the assessed cognitive activity, such as the hippocampus, PFC, and parietal cortex. In addition, proteins related to synaptic neuroplasticity, such as synapsin I, synaptophysin, and PSD95 should be measured.

## Conclusion

In conclusion, the caffeine metabolite 1-MX is physiologically active and increases memory, neurotransmitter levels, neuroplasticity and several markers of neuronal health. Our study demonstrates that 1-MX is a new nootropic dietary ingredient supporting brain health and performance.
